# Prehistoric Art

**Published:** 1886-03

**Authors:** 


### PREHISTORIC ART.

We have received from Dr. Van Marter the contents of the oldest Etrurian tomb yet opened in Italy, at Capadimonti, which was referred to in his article on page 59 of last month's issue. It includes a piece of ancient Etrurian dentistry one hundred years older than any before discovered, with a number of articles for personal adornment, bronzes, vases, etc. A later number will contain a full account of this very interesting and valuable archæological discovery.

